# A Multimodal Multi‐task Model Integrating Scalp Imaging, Lipidomics, and Microbiome Data for Scalp Condition and Social Stigma: An Algorithm Development and Validation Study Across Multi‐ethnic Cohorts

**DOI:** 10.1111/jocd.71217

**Published:** 2026-10-02

**Authors:** Jiyeon Oh, Soeun Kim, Taelim Choi, Su Min Son, Kyeongmo Lim, Kyong‐Oh Shin, So Min Kang, Kyung‐Eun Lee, Kyungho Park, Dasom Jeon, Hyo Je Ahn, Su Bin Jin, Da‐Ryung Jung, Hye‐Been Kim, Seoyeon Kyung, Dong‐Geol Lee, Jae‐Ho Shin, Seunghyun Kang, Chunho Park, Selin Woo, HyungWoo Jo, Dong Keon Yon

**Affiliations:** ^1^ Center for Digital Health, Medical Science Research Institute Kyung Hee University College of Medicine Seoul South Korea; ^2^ Department of Medicine Kyung Hee University College of Medicine Seoul South Korea; ^3^ Department of Precision Medicine Kyung Hee University College of Medicine Seoul South Korea; ^4^ COSMAX BTI R&I Center Seongnam South Korea; ^5^ Department of Applied Biosciences Kyungpook National University Daegu South Korea; ^6^ LaSS Lipid Institute (LLI), LaSS Inc. Chuncheon South Korea; ^7^ Department of Food Science and Nutrition Convergence Program of Material Science for Medicine and Pharmaceutics, Korean Institute of Nutrition, Hallym University Chuncheon South Korea; ^8^ KBI Korea Biomedical Research Institute Seongnam South Korea; ^9^ NGS Core Facility Kyungpook National University Daegu South Korea; ^10^ COSMAX R&I Center Seongnam South Korea; ^11^ Department of Pediatrics, Kyung Hee University Medical Center Kyung Hee University College of Medicine Seoul South Korea

To the Editor,

Scalp health carries both physical and psychosocial burden, such as social stigma, yet objective, standardized assessment tools remain limited [1]. Although deep learning methods have shown strong performance in medical image analysis and scalp disorder classification [2, 3], scalp‐specific models integrating multi‐omics and behavioral data with validation across diverse ethnic cohorts remain lacking. To address these gaps, we developed and externally validated a multimodal, multitask deep learning framework integrating scalp imaging, lipidomics, and microbiome data for scalp condition and social stigma assessment across Korean, White, and Black individuals.

We obtained scalp images, questionnaire‐based metadata, microbiome profiles, and lipidomic data from a total of 363 participants, comprising 305 Korean, 29 White, and 29 Black individuals, enrolled by the Korea Biomedical Research Institute between February 2025 and June 2025, following the principles of the Declaration of Helsinki. Written informed consent was obtained from all individuals prior to enrollment. This study was approved by the Ethics Committee of Korea Biomedical Research Institute Institutional Review Board (F‐2025‐008‐1) [4].

The Korean cohort was randomly partitioned into development and internal test sets in a 7:3 ratio, with stratification based on the presence of any hair‐related condition. Fivefold stratified cross‐validation was subsequently performed within the development set using the same stratification criterion, whereas the White and Black cohorts were reserved exclusively for external validation. Four hair‐related outcomes (dryness, oiliness, erythema, and dandruff) and the Social Stigma Clinical Index (SSCI) were assessed using standardized protocols, and scalp images were preprocessed as described in Appendix S1. Image features were extracted using a self‐supervised contrastive learning framework (SimCLR) based on a ResNet‐50 backbone [5], followed by multitask prediction of hair‐related outcomes and ordinal SSCI using a COnsistent RAnk Logits (CORAL)‐based approach [6]. Structured metadata were modeled using a multilayer perceptron with multitask learning after feature preprocessing and selection (Table S1). Microbiome and lipidomic data were integrated using a Phenotype‐Aware Parallel Representation for Integrative omiC Analysis (PAPRICA)‐based parallel autoencoder [7]. PAPRICA was selected as the omics‐integration component because its parallel architecture enables modality‐specific representation learning while capturing shared cross‐omics information in a low‐dimensional latent space. This design was suited to the present multimodal framework, in which the resulting omics representations were subsequently integrated with independently derived image embeddings and structured metadata for downstream prediction (Figure 1). Model performance was evaluated using the area under the precision‐recall curve (AUPRC) and the area under the receiver operating characteristic curve (AUROC) for classification outcomes, and mean absolute error (MAE), root mean squared error (RMSE), quadratic weighted kappa (QWK), and Spearman correlation for SSCI. Model interpretability was assessed using Shapley Additive Explanations (SHAP) analysis.

**FIGURE 1:**
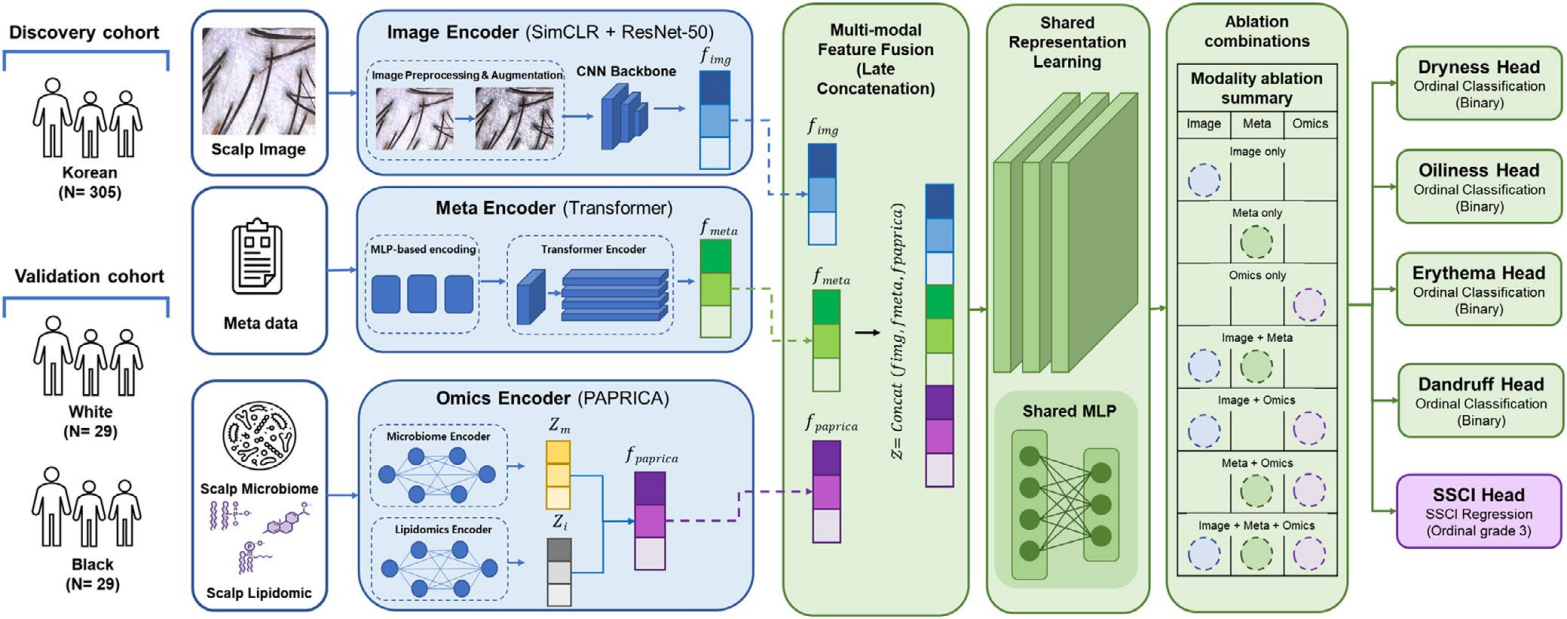
Architecture of the proposed multimodal deep learning framework for scalp condition assessment. CNN, convolutional neural network; MLP, multilayer perceptron; PAPRICA, Phenotype‐Aware Parallel Representation for Integrative omiC Analysis; SimCLR, self‐supervised contrastive learning framework; SSCI, Social Stigma Clinical Index.

Of 363 individuals, the mean age was 47.60 years (standard deviation, 12.66), and 17.08% (*n* = 62) were female (Table S2). Among all evaluated models, the fully integrated multimodal model incorporating metadata, image, and omics‐derived representations was selected as the final model based on its overall performance across the development and internal test datasets. In the Korean training set, it achieved an AUROC of 0.788 (95% confidence intervals, 0.742–0.836) and AUPRC of 0.766 (0.703–0.819) for any hair‐related condition, with an AUROC of 0.928 and AUPRC of 0.927 in the internal test set (Table 1). Performance was particularly strong for erythema (AUROC, 0.971) and dandruff (AUROC, 0.943). For SSCI, the model exhibited moderate ordinal discrimination (MAE, 0.47; RMSE, 0.81; QWK, 0.53; Spearman, 0.55). In external validation, acceptable performance was maintained in the White cohort (AUROC, 0.708 for any hair condition) but attenuated in the Black cohort (AUROC, 0.581 for any hair condition), reflecting cross‐ethnic generalization challenges (*n* = 29 per group). According to SHAP analysis, image‐derived representations capturing sebum‐related scalp characteristics were among the most influential contributors, together with hair‐combing frequency and a PAPRICA‐derived latent feature (FUSPAP_001), for scalp‐related outcomes (Figure S1). In contrast, hair dryness level and another latent feature (FUSPAP_005) were the dominant contributors to SSCI prediction. Detailed mappings are provided in Tables S3–S5.

**TABLE 1:** A comparative analysis of the predictive performance of models using training and test datasets from Korean and external validation datasets from White and Black participants.

	Model	Any hair condition^a^		Mean^b^		SSCI				Dryness		Oiliness		Erythema		Dandruff	
		AUROC	AUPRC	AUROC	AUPRC	MAE	RMSE	QWK	Spearman	AUROC	AUPRC	AUROC	AUPRC	AUROC	AUPRC	AUROC	AUPRC
Korean (train), mean (95% CI)	Meta dataset	0.533 (0.473 to 0.593)	0.450 (0.390 to 0.532)	0.509 (0.434 to 0.588)	0.169 (0.130 to 0.221)	0.79 (0.73 to 0.85)	0.99 (0.94 to 1.05)	0.08 (−0.01 to 0.16)	0.09 (−0.02 to 0.14)	0.538 (0.364 to 0.700)	0.152 (0.031 to 0.284)	0.508 (0.447 to 0.571)	0.325 (0.263 to 0.398)	0.472 (0.246 to 0.697)	0.053 (0.017 to 0.143)	0.519 (0.442 to 0.590)	0.144 (0.109 to 0.192)
	Image dataset	0.742 (0.691 to 0.790)	0.694 (0.616 to 0.770)	0.789 (0.749 to 0.830)	0.460 (0.357 to 0.552)	0.87 (0.80 to 0.95)	1.14 (1.10 to 1.20)	0.09 (−0.01 to 0.19)	0.10 (−0.01 to 0.20)	0.721 (0.600 to 0.835)	0.196 (0.054 to 0.370)	0.714 (0.657 to 0.771)	0.526 (0.432 to 0.613)	0.977 (0.952 to 0.996)	0.631 (0.317 to 0.917)	0.746 (0.661 to 0.827)	0.488 (0.340 to 0.613)
	Microbiome dataset	0.558 (0.498 to 0.619)	0.482 (0.407 to 0.560)	0.513 (0.444 to 0.579)	0.167 (0.136 to 0.208)	0.87 (0.82 to 0.93)	1.04 (0.99 to 1.09)	−0.10 (−0.18 to −0.02)	−0.13 (−0.24 to −0.02)	0.500 (0.355 to 0.645)	0.098 (0.030 to 0.218)	0.546 (0.480 to 0.613)	0.351 (0.282 to 0.430)	0.455 (0.248 to 0.670)	0.039 (0.018 to 0.057)	0.551 (0.473 to 0.626)	0.178 (0.118 to 0.244)
	Lipidomic dataset	0.827 (0.782 to 0.872)	0.842 (0.793 to 0.885)	0.713 (0.657 to 0.771)	0.323 (0.274 to 0.371)	0.81 (0.75 to 0.87)	1.00 (0.95 to 1.05)	0.05 (−0.04 to 0.13)	0.06 (−0.5 to 0.16)	0.790 (0.719 to 0.857)	0.176 (0.064 to 0.301)	0.725 (0.669 to 0.782)	0.566 (0.471 to 0.650)	0.492 (0.304 to 0.688)	0.043 (0.020 to 0.062)	0.839 (0.784 to 0.897)	0.505 (0.358 to 0.635)
	Fusion	0.867 (0.827 to 0.904)	0.867 (0.822 to 0.906)	0.742 (0.689 to 0.803)	0.312 (0.277 to 0.350)	0.81 (0.74 to 0.88)	1.06 (0.10 to 1.12)	0.02 (−0.07 to 0.11)	0.03 (−0.07 to 0.14)	0.852 (0.798 to 0.901)	0.169 (0.091 to 0.249)	0.740 (0.687 to 0.792)	0.551 (0.450 to 0.642)	0.507 (0.324 to 0.704)	0.043 (0.019 to 0.063)	0.867 (0.823 to 0.912)	0.486 (0.347 to 0.612)
	Meta + Image	0.807 (0.761 to 0.851)	0.800 (0.744 to 0.850)	0.898 (0.872 to 0.923)	0.696 (0.596 to 0.781)	0.46 (0.39 to 0.52)	0.81 (0.73 to 0.88)	0.53 (0.44 to 0.61)	0.56 (0.47 to 0.64)	0.943 (0.887 to 0.991)	0.605 (0.339 to 0.844)	0.753 (0.699 to 0.808)	0.586 (0.490 to 0.677)	0.964 (0.911 to 0.999)	0.823 (0.532 to 1.000)	0.931 (0.895 to 0.974)	0.771 (0.652 to 0.871)
	Meta + Fusion	0.828 (0.784 to 0.871)	0.802 (0.739 to 0.857)	0.741 (0.691 to 0.798)	0.315 (0.267 to 0.366)	0.47 (0.41 to 0.54)	0.81 (0.74 to 0.88)	0.51 (0.43 to 0.60)	0.54 (0.45 to 0.62)	0.689 (0.540 to 0.845)	0.129 (0.047 to 0.212)	0.745 (0.692 to 0.796)	0.570 (0.471 to 0.666)	0.718 (0.568 to 0.869)	0.115 (0.033 to 0.228)	0.809 (0.748 to 0.874)	0.440 (0.308 to 0.570)
	Image + Fusion	0.798 (0.752 to 0.844)	0.775 (0.710 to 0.830)	0.871 (0.831 to 0.910)	0.636 (0.513 to 0.750)	0.83 (0.76 to 0.89)	1.05 (0.99 to 1.11)	0.06 (−0.02 to 0.15)	0.08 (−0.02 to 0.18)	0.901 (0.814 to 0.980)	0.524 (0.231 to 0.754)	0.743 (0.690 to 0.798)	0.573 (0.472 to 0.662)	0.917 (0.821 to 0.999)	0.666 (0.296 to 0.999)	0.926 (0.885 to 0.974)	0.790 (0.685 to 0.888)
	Meta + Image + Fusion	0.788 (0.742 to 0.836)	0.766 (0.703 to 0.819)	0.893 (0.861 to 0.922)	0.676 (0.568 to 0.764)	0.47 (0.40 to 0.53)	0.81 (0.73 to 0.88)	0.53 (0.45 to 0.62)	0.55 (0.47 to 0.63)	0.918 (0.843 to 0.983)	0.537 (0.249 to 0.753)	0.771 (0.719 to 0.823)	0.612 (0.514 to 0.703)	0.958 (0.898 to 0.999)	0.778 (0.495 to 0.999)	0.925 (0.884 to 0.971)	0.779 (0.662 to 0.884)
Korean (test)	Meta only	0.626	0.600	0.617	0.254	0.80	1.03	0.04	0.05	0.777	0.349	0.582	0.339	0.505	0.113	0.603	0.214
	Image only	0.861	0.879	0.79	0.463	0.91	1.20	0.00	0.00	0.832	0.299	0.688	0.370	0.866	0.689	0.774	0.494
	Microbiome	0.586	0.623	0.563	0.256	0.89	1.05	−0.1	−0.14	0.570	0.254	0.621	0.390	0.442	0.096	0.618	0.285
	Lipidomic	0.889	0.916	0.719	0.314	0.82	1.01	0.04	0.02	0.805	0.322	0.715	0.446	0.570	0.150	0.787	0.338
	Fusion	0.926	0.940	0.727	0.347	0.88	1.06	−0.06	−0.08	0.817	0.34	0.746	0.549	0.526	0.145	0.82	0.353
	Meta + Image	0.903	0.904	0.907	0.765	0.37	0.75	0.64	0.65	0.98	0.892	0.752	0.576	0.956	0.913	0.94	0.68
	Meta + Fusion	0.906	0.903	0.749	0.379	0.36	0.73	0.66	0.66	0.754	0.294	0.757	0.523	0.631	0.235	0.854	0.463
	Image + Fusion	0.91	0.908	0.908	0.778	0.80	0.98	0.11	0.13	0.966	0.844	0.761	0.603	0.967	0.918	0.94	0.745
	Meta + Image + Fusion	0.928	0.927	0.919	0.802	0.36	0.75	0.65	0.66	0.973	0.888	0.791	0.658	0.971	0.921	0.943	0.74
White	Meta + Image + Fusion	0.708	0.696	0.741	0.587	0.82	1.15	0.00	0.00	—	—	0.678	0.507	0.598	0.442	0.947	0.813
Black	Meta + Image + Fusion	0.581	0.688	0.648	0.674	0.90	1.20	0.10	0.09	—	—	0.505	0.626	—	—	0.792	0.722

*Note:* Fusion indicates the integration of microbiome and lipidomic data. Blue highlight denotes the final selected model. CIs were estimated using bootstrap resampling (2000 iterations). — indicates that performance could not be estimated because no cases of the corresponding outcome were observed.

Abbreviations: AUPRC, area under the precision‐recall curve; AUROC, area under the receiver operating characteristic curve; CIs, confidence intervals; MAE, mean absolute error; QWK, quadratic weighted kappa; RMSE, root mean squared error; SSCI, Social Stigma Clinical Index.

^a^ Any hair condition indicates the presence of ≥ 1 hair‐related condition.

^b^ Mean represents the average performance across all four hair‐related outcomes.

Compared to previous multimodal approaches for skin and scalp assessment, the present study is distinctive in simultaneously predicting objective scalp conditions and patient‐reported social stigma within a unified multitask framework, with external validation across three ethnic cohorts. While the fully integrated model showed strong overall performance, the competitive results of the model integrating metadata and imaging suggest that these modalities carry substantial predictive information, while omics may provide complementary rather than uniformly additive benefit. SHAP analysis supports clinical plausibility: sebum‐related image features and combing frequency were key predictors of scalp outcomes [8]. The moderate SSCI prediction accuracy, despite its subjective nature, suggests that objectively measured scalp features partially reflect patient‐perceived psychosocial burden, underscoring the value of integrating patient‐centered outcomes with biological measures in scalp health assessment. Beyond classification performance, the multimodal design enables flexible assessment using readily accessible image and metadata, with omics providing complementary biological information when available.

Performance decline in external cohorts, particularly among Black participants, likely reflects limited validation sample size, differences in scalp physiology, and behavioral heterogeneity across cohorts (Table S2). In particular, reduced erythema visibility on darker skin tones and differences in hair texture and scalp coverage may have further contributed to distributional shifts not captured in the training data. Although large‐scale multiethnic recruitment is challenging given the resource‐intensive nature of multimodal data collection, future work should prioritize larger cohorts spanning diverse skin tones and hair types under standardized acquisition protocols, with clinical validation. Future longitudinal studies are warranted to determine whether this framework can detect subtle or early‐stage changes before overt clinical manifestations. In resource‐limited settings, the “Meta + Image” model alone (AUROC 0.807 on the Korean training set) may serve as a practical alternative to the full pipeline.

We present a multimodal, multitask deep learning framework for simultaneous prediction of scalp conditions and SSCI, showing strong internal performance while highlighting challenges in cross‐ethnic generalizability. Further validation in larger, more diverse cohorts remains essential for clinical translation.

## Author Contributions

D.K.Y. had full access to all of the data in the study and took responsibility for the integrity of the data and the accuracy of the data analysis. All authors approved the final version of the manuscript before submission. Study concept and design: J.O., Soeun Kim, T.C., S.M.S., K.L., K.‐O.S., S.M.K., S.W., H.J., and D.K.Y.; acquisition, analysis, or interpretation of data: J.O., Soeun Kim, T.C., S.W., and D.K.Y.; drafting of the manuscript: J.O., Soeun Kim, T.C., S.M.S., K.L., K.‐O.S., S.M.K., S.W., H.J., and D.K.Y.; critical revision of the manuscript for important intellectual content: all authors; statistical analysis: J.O., Soeun Kim, and T.C.; study supervision: D.K.Y. D.K.Y. supervised the study and served as a guarantor. The corresponding author attests that all listed authors meet the authorship criteria and that no one meeting the criteria has been omitted.

## Funding

This research was supported by COXMAX BTI Inc. In addition, this research was partially supported by the Ministry of Science and ICT (IITP‐2026‐RS‐2024‐00438239) and the Ministry of Health & Welfare (RS‐2025‐02220492), Republic of Korea. The study steering committee led the cohort design, with support from the sponsor (COXMAX BTI Inc.). The study steering committee compiled and analyzed the data. The study steering committee vouches for the accuracy and completeness of the data and for the fidelity of the cohort to the protocol.

## Conflicts of Interest

The authors declare no conflicts of interest.

## Supporting information


**Appendix S1:** Supplementary methods.
**Figure S1:** SHAP‐based feature importance analysis for scalp issue prediction across image, metadata, and fusion models. Detailed descriptions of the features included in the fusion model are provided in Tables S1 and S2. SSCI, Social Stigma Clinical Index; SHAP, Shapley Additive Explanation.
**Table S1:** Detailed definitions of study variables.
**Table S2:** Baseline characteristics of the study population in the discovery cohort from Korea and external validation cohorts of White and Black participants.
**Table S3:** A comparative analysis of the predictive performance of models using training and test datasets from Korean and external validation datasets from White and Black participants.
**Table S4:** Mapping of fusion latent variables to original microbiome and lipid features for hair‐related outcome.
**Table S5:** Mapping of fusion latent variables to original microbiome and lipid features for SSCI outcome.

## Data Availability

The data and workflow code underlying this study are available from the corresponding author upon reasonable request, subject to institutional approval, ethical considerations, and applicable data‐use agreements. Participant‐level raw data are not publicly available because they include potentially identifiable scalp images and linked clinical and multi‐omics information. Requests should be directed to the corresponding author at yonkkang@gmail.com.
