# Supplementary material for: A Multimodal Multi‐task Model Integrating Scalp Imaging, Lipidomics, and Microbiome Data for Scalp Condition and Social Stigma: An Algorithm Development and Validation Study Across Multi‐ethnic Cohorts
Source: J Cosmet Dermatol. 2026 Oct 2;25(10):e71217. doi: 10.1111/jocd.71217 (PMC13633382; doi:10.1111/jocd.71217)
Supplement: Supplementary file 1 [file JOCD-25-e71217-s001.docx]

**Supplementary Material**

Letter

**A multimodal multi-task model integrating scalp imaging, lipidomics, and microbiome data for scalp condition and social stigma: an algorithm development and validation study across multi-ethnic cohorts**

**Running title:** Multimodal model for scalp condition and stigma

Jiyeon Oh^1,2#^, Soeun Kim^1,3#^, Taelim Choi^,1,3#^, Su Min Son^4#^, Kyeongmo Lim^5#^, Kyong-Oh Shin^6#^, So Min Kang^7#^, Kyung-Eun Lee^4^, Kyungho Park^6^, Dasom Jeon^7^, Hyo Je Ahn^7^, Su Bin Jin^7^, Da-Ryung Jung^8^, Hye-Been Kim^4^, Seoyeon Kyung^4^, Dong-Geol Lee^4^, Jae-ho Shin^5^, Seunghyun Kang^9^, Chunho Park^4^, Selin Woo^1,2*^, HyungWoo Jo^4^*, Dong Keon Yon^1,2,3,10*^

^#^These authors contributed equally.

***** These authors contributed equally.

**Corresponding author**

**Dong Keon Yon**, MD, PhD, FACAAI, FAAAAI, ATSF (lead contact)

Department of Pediatrics, Kyung Hee University College of Medicine, 23 Kyungheedae-ro, Dongdaemun-gu, Seoul 02447, South Korea

Email: yonkkang@gmail.com

**Contents**

[**1. Supplementary methods**	3](#_Toc240989138)

[**1.1 Multi-omics profiling of scalp samples**	3](#_Toc240989139)

[**1. 2 Scalp imaging acquisition**	5](#_Toc240989140)

[**1.3 Image preprocessing**	6](#_Toc240989141)

[**1.4 Meta data collection and processing**	7](#_Toc240989142)

[**1.5 Image-based representation learning and multi-task prediction model**	9](#_Toc240989143)

[**1.6 Meta-data modeling using a multilayer perceptron with multi-task learning**	11](#_Toc240989144)

[**1.7 PAPRICA-based multimodal latent representation learning**	12](#_Toc240989145)

[**1.9 Evaluation metrics**	14](#_Toc240989146)

[**1.10 Explainable artificial intelligence**	16](#_Toc240989147)

[**2. Supplementary Figure**	17](#_Toc240989148)

[**3. Supplementary Tables**	18](#_Toc240989149)

[**Table S1.** Detailed definitions of study variables	18](#_Toc240989150)

[**Table S2.** Baseline characteristics of the study population in the discovery cohort from Korea and external validation cohorts of white and black participants.	22](#_Toc240989151)

[**Table S3.** A comparative analysis of the predictive performance of models using training and test datasets from Korean and external validation datasets from White and Black participants.	27](#_Toc240989152)

[**Table S4.** Mapping of fusion latent variables to original microbiome and lipid features for hair-related outcome.	29](#_Toc240989153)

[**Table S5.** Mapping of fusion latent variables to original microbiome and lipid features for SSCI outcome.	36](#_Toc240989154)

[**4 Reference**	43](#_Toc240989155)

# **1. Supplementary methods**

## **1.1 Multi-omics profiling of scalp samples**

Participants were instructed to complete shampooing by midnight (24:00) the evening prior to sampling, allowing sufficient recovery time for the scalp microbiome. For sample collection, the hair was parted using a comb to maximize scalp exposure, and microbial samples were collected from an approximately 15 cm × 15 cm area near the crown of the scalp using sterile collection swabs (Noble Bio, Hwaseong, South Korea). Each swab was applied with firm, consistent pressure in a back-and-forth motion for 3 minutes, and collected samples were stored frozen at −20°C until DNA extraction.

Collected scalp swab samples were then subjected to total genomic DNA extraction using the DNeasy PowerSoil Pro Kit (QIAGEN, Hilden, Germany) according to the manufacturer’s instructions. DNA quantity and quality were assessed prior to library preparation. To achieve species-level taxonomic resolution, long-read amplicon sequencing was performed on the PacBio Sequel IIe platform (Pacific Biosciences, Menlo Park, CA, USA). The near full-length bacterial 16S rRNA gene (V1–V9) and the full-length fungal ITS (ITS1–5.8S–ITS2) regions were amplified and sequenced. Libraries were prepared following the SMRTbell protocol, and circular consensus sequencing (CCS, HiFi reads) was generated.

Sequencing data were processed using QIIME 2 (version 2025.4). Primer removal and demultiplexing were conducted using q2-cutadapt, followed by denoising with DADA2 in CCS mode to obtain amplicon sequence variants (ASVs). Taxonomic assignment was performed using a naïve Bayes classifier trained on the Genome Taxonomy Database (GTDB, r220) for bacterial 16S sequences and the UNITE database for fungal ITS sequences. The resulting ASV table was used to generate sample-level taxonomic abundance profiles.

In parallel, lipidomic profiling was conducted on the same scalp samples to comprehensively characterize the biochemical environment of the scalp. Total lipids were extracted using a modified Bligh and Dyer method, as previously described.^1^ A mixture of class-specific internal standards was added prior to extraction to ensure accurate quantification. After phase separation, the organic layer was collected, dried under nitrogen, and reconstituted for analysis.

Lipidomic profiling was performed using liquid chromatography–tandem mass spectrometry (LC–MS/MS) on a SCIEX 5500 QTRAP system (AB Sciex, Framingham, MA, USA) equipped with an electrospray ionization source. Lipid species were separated on a reverse-phase C18 column and analyzed in multiple reaction monitoring mode under both positive and negative ionization conditions. The targeted lipid panel included major lipid classes such as triglycerides, wax esters, phospholipids, sphingolipids, cholesterol-related lipids, and free fatty acids. Data acquisition and quantification were conducted using Analyst 1.7.1 and MultiQuant software (AB Sciex, Framingham, MA, USA). Lipid levels were normalized to total protein content measured by a BCA assay and expressed as pmol or nmol per mg protein.

## **1. 2 Scalp imaging acquisition**

Scalp condition was assessed using the ASW-300 (Aram Huvis Co., Ltd., Daejeon, South Korea) at 30× magnification. Images were acquired in a standardized manner across key scalp regions, including the crown and temporal areas, to ensure objective and consistent documentation. All images were analyzed by a trained evaluator. Five representative indicators of scalp condition were assessed: dryness, oiliness, erythema, and dandruff. Each parameter was graded on a 4-point scale ranging from 0 (none) to 3 (severe), where 0 indicated no observable symptoms, 1 indicated mild involvement, 2 indicated moderate involvement, and 3 indicated severe involvement.

## **1.3 Image preprocessing**

In this study, image preprocessing was performed using contrast-limited adaptive histogram equalization (CLAHE), Telea-based inpainting, and bilateral filtering. First, images were converted to the LAB color space, and CLAHE was applied to the luminance channel to enhance local contrast while limiting over-amplification.^2^ Next, high-intensity regions in the HSV color space were defined as glare, and Telea-based inpainting was applied to correct illumination-induced artifacts and information loss.^3^ Subsequently, bilateral filtering was applied to suppress noise while preserving structural boundaries and fine tissue details.^4^ Finally, all images were resized to 224 × 224 pixels to ensure consistency for model input.

## **1.4 Meta data collection and processing**

A self-administered structured questionnaire was used to assess scalp- and hair-related lifestyle habits. The questionnaire comprised 42 items across five domains: basic demographics (sex, age, height, weight); hair care habits (frequency of bleaching, dyeing, perming, and cutting; shampooing frequency and timing; frequency of heat tool use, hat wearing, and hair tying); general lifestyle factors (sleep duration, smoking, alcohol consumption, and perceived stress); self-perceived scalp condition (scalp type, scalp concerns, hair loss status, and dandruff characteristics); and scalp/hair product usage (product types used, medication use, and professional scalp care experience). Items were presented in single- or multiple-choice formats, and frequency-based scales were applied to selected items to enable quantitative comparison of lifestyle habits across participants.

Subjective symptoms related to the scalp, hair, and hair loss were quantified using a Visual Analogue Scale, presented as a continuous scale from 0 (not at all) to 10 (extremely) and completed via self-report. Items were organized into three domains: scalp (8 items), hair (10 items), and hair loss (5 items), yielding a total of 23 items, with higher scores indicating greater symptom severity.

Perceived social stigma associated with scalp and hair conditions was assessed using the Stigma Scale for Chronic Illnesses 8-item version (SSCI-8), a validated self-report instrument comprising six items assessing enacted stigma and two items assessing internalized stigma.^5^ Each item was rated according to the frequency of stigma-related experiences on a 5-point Likert scale ranging from 1 (never) to 5 (always). The eight item scores were summed to generate a total score ranging from 8 to 40, with higher scores indicating greater perceived stigmatization.^5^

Feature selection was conducted to reduce dimensionality and mitigate the risk of overfitting given the large number of collected variables. To achieve this, both L1-regularized logistic regression (LASSO) and a random forest–based importance approach were applied to enhance model interpretability.^6^ Variables with non-zero coefficients in the LASSO model and those exceeding the median importance threshold in the random forest model were selected.^6^ This dual approach enabled the identification of relevant predictors by combining linear sparsity-based selection with non-linear importance ranking.

A total of 31 features were retained for model development, comprising both continuous and categorical variables. Continuous variables included age in years; anthropometric measures, namely height in cm, weight in kilograms, and body mass index in kg/m²; and vital signs, including systolic and diastolic blood pressure in mmHg and pulse rate in beats per minute. Categorical variables encompassed clinical characteristics, scalp condition, hair care practices, cosmetic behaviors, lifestyle factors, and life-course variables. Detailed definitions of all variables and their categories are provided in **Table S1**.

## **1.5 Image-based representation learning and multi-task prediction model**

Preprocessed images were first used to construct a self-supervised contrastive learning-based pretraining model. Specifically, the SimCLR framework was employed, in which two augmented views of the same image were generated and trained to yield similar representations, thereby facilitating the learning of generalizable visual features.^7^ A ResNet50 architecture served as the backbone, and feature representations were optimized using a contrastive loss function.^8,9^

The pretrained model was subsequently used as the foundation for the image-based prediction model. Preprocessed images were converted into fixed-size input tensors and passed through the network, where high-dimensional embeddings were extracted from intermediate layers and propagated through fully connected layers to generate final predictions.

Model outputs were expressed as probability scores for each hair-related outcome, representing the likelihood of the presence of each condition. In addition, a multi-task learning framework was adopted, incorporating ordinal prediction of Social Stigma Clinical Index using a COnsistent RAnk Logits (CORAL)-based output, along with an auxiliary binary output for high-risk classification.^10,11^ Accordingly, the model functioned not only as a classifier but also as a feature extractor, producing latent visual representations reflective of scalp conditions.

Model training was conducted using the training dataset, with the optimal model selected based on validation performance. The final trained model was subsequently applied to the internal test set and external validation dataset without further updating, enabling an unbiased evaluation of generalizability.

Furthermore, image embeddings extracted from the penultimate layer of the backbone network were separately stored and used as inputs for downstream multimodal integration.^12^ This design enabled the image model to function both as an independent predictive model and as a representation learning module integrated with meta-data and Phenotype-Aware Parallel Representation for Integrative omiC Analysis (PAPRICA)-derived latent features.^13^

## **1.6 Meta-data modeling using a multilayer perceptron with multi-task learning**

Preprocessed meta-data consisted of structured input variables, and continuous features were standardized using a StandardScaler prior to model training.^14^ All preprocessing procedures were performed exclusively on the training data and subsequently applied to the validation and test datasets to prevent data leakage. The model was constructed based on a multilayer perceptron (MLP) architecture, in which input features were transformed into latent representations through nonlinear transformations.^15^ These representations were subsequently passed through fully connected layers to generate final predictions.

The output was designed within a multi-task learning framework, consistent with the image model. Specifically, the model produced binary probability estimates for each hair-related outcome, along with ordinal predictions of the SSCI using a CORAL-based output. In addition, an auxiliary binary output was included to identify high-risk cases.

Model training was conducted using the training dataset, with optimization performed to minimize a multi-task objective function. The optimal model was selected based on validation performance. The final trained model was then applied to the internal test set and external validation dataset without further updating, enabling an unbiased evaluation of generalizability.

## **1.7 PAPRICA-based multimodal latent representation learning**

A PAPRICA-based fusion autoencoder model was developed to integrate complementary information from microbiome and lipidomic data. The PAPRICA framework learns a shared latent representation by jointly modeling multiple omics modalities while preserving modality-specific information.^13^ First, the two datasets were aligned and matched based on a common identifier, and each modality was constructed as a separate input matrix. The model comprised modality-specific encoders and decoders for microbiome and lipidomic data, respectively. Each encoder projected high-dimensional input features into a shared latent space, while each decoder was trained to reconstruct the original input from the corresponding latent representation.^13^

Model training involved the joint optimization of two objectives. A reconstruction loss was used to preserve the intrinsic structure of each modality, and a coupling loss was introduced to encourage alignment between the latent representations derived from microbiome and lipidomic data.^13^ Through this joint optimization, the model retained modality-specific characteristics while capturing shared patterns across omics data, resulting in an integrated latent representation.^16^

After training, latent representations from the microbiome and lipidomic encoders were extracted for each sample, and a fused latent representation was obtained by averaging the two modality-specific vectors.^13^ These low-dimensional latent features were stored as a new feature set and subsequently used as inputs for downstream multimodal integration, where they were combined with image embeddings and meta-data for final prediction of hair-related outcomes and SSCI using a CORAL-based ordinal output.

**1.8 Multimodal integration using feature-level late fusion**

A multimodal prediction model was constructed by integrating image embeddings extracted from the image model, structured variables used in the meta model, and latent features generated by the PAPRICA model into a single feature set. All modalities were aligned and matched using a common identifier, and only samples available across all modalities were retained through inner-join merging. This procedure was applied consistently to the training, internal test, and external validation datasets.

The features from each modality were concatenated into a single vector and used as inputs to the downstream multi-task prediction model, which simultaneously predicted hair-related outcomes, ordinal SSCI levels using a CORAL-based output, and high-risk classification.^10^ A feature-level late fusion strategy was adopted, whereby modality-specific representations were learned independently and subsequently integrated via concatenation for final prediction, enabling the preservation of modality-specific information while leveraging complementary signals across modalities.

## **1.9 Evaluation metrics**

In this study, we developed a multi-task learning framework to simultaneously address two prediction tasks: (i) binary classification of four hair-related outcomes and (ii) ordinal classification of the SSCI. Each task was evaluated using performance metrics appropriate to its outcome structure.

Each hair-related endpoint was formulated as an independent binary classification task. Model performance was assessed using the area under the receiver operating characteristic curve (AUROC) and the area under the precision–recall curve (AUPRC).^14^ AUROC reflects the model’s overall discriminative ability across classification thresholds, whereas AUPRC provides a more informative evaluation in the presence of class imbalance by capturing the trade-off between precision and recall. Performance was reported for each outcome individually and summarized using the mean AUROC and mean AUPRC across tasks, along with a composite metric (i.e., any hair condition) representing the probability that at least one hair-related condition was present.

The SSCI outcome was treated as an ordinal variable with three categories reflecting increasing levels of social stigma (1–8, 9–16, and ≥17).14 To model its ordinal nature, a CORAL-based framework was employed, which reformulates ordinal classification into K−1 ordered binary tasks with shared weights.^10^ The model outputs cumulative logits that enforce consistency in the predicted ordering across categories. Model performance was evaluated using metrics that capture both prediction error and rank consistency. Mean absolute error (MAE) and root mean squared error (RMSE) were used to quantify prediction error, with lower values indicating better performance. Agreement between predicted and observed categories was assessed using the quadratic weighted kappa (QWK), which accounts for the ordinal structure, while the Spearman rank correlation coefficient was used to evaluate monotonic association, with higher values indicating better preservation of rank order.^17^

Robustness of model performance was assessed using cross-validation in conjunction with bootstrap-based confidence intervals.^14^ All preprocessing steps, including imputation and scaling, were performed exclusively within each training fold to prevent data leakage and ensure an unbiased evaluation of model generalizability.

## **1.10 Explainable artificial intelligence**

Explainability for image-based predictions was achieved using Gradient-weighted Class Activation Mapping (Grad-CAM), which provides visual explanations of model decisions. Grad-CAM generates class-specific activation maps by leveraging gradients flowing into the final convolutional layers, thereby highlighting regions of the input image that contribute most strongly to the prediction.^9^ In this study, Grad-CAM was used to identify salient scalp regions associated with both hair-related outcomes and SSCI prediction, enabling qualitative assessment of whether the model focused on clinically relevant patterns.

Model interpretability for meta-data and PAPRICA-derived features was assessed using SHapley Additive exPlanations (SHAP). SHAP quantifies the contribution of each feature to individual predictions based on cooperative game theory, allowing both global and local interpretation of model behavior. For meta-data, SHAP values were directly computed on the original input variables to identify key predictors.^14^

Interpretation of PAPRICA-derived latent features was further extended to bridge the gap between latent representations and original omics variables. Specifically, the contribution of latent features identified by SHAP was examined by mapping them back to their corresponding input features, enabling identification of biologically relevant microbiome and lipidomic components underlying the model predictions.^18^

# **2. Supplementary Figure**

**Figure S1.** **SHAP-based feature importance analysis for scalp issue prediction across image, metadata, and fusion models.** Detailed descriptions of the features included in the fusion model are provided in **Tables S1** and **S2**.

*Abbreviations*: SSCI, Social Stigma Clinical Index Social Stigma Clinical Index; SHAP, Shapley Additive Explanation

# **3. Supplementary Tables**

## **Table S1.** Detailed definitions of study variables

| **Variables** | **Type** | **Description** |
| --- | --- | --- |
| **Sex** | 2 categories | - Male |
|  |  | - Female |
| Age | Numerical | - years |
| Height | Numerical | - cm |
| Weight | Numerical | - kg |
| BMI | Numerical | - kg/m² |
| SBP | Numerical | - mmHg |
| DBP | Numerical | - mmHg |
| Pulse | Numerical | - Beats per minute |
| Alopecia status | 4 categories | - No suspected alopecia |
|  |  | - Suspected alopecia |
|  |  | - Currently experiencing alopecia |
|  |  | - Uncertain |
| Scalp type | 4 categories | - Oily |
|  |  | - Normal |
|  |  | - Dry |
|  |  | - Not sure |
| Severity of scalp odor | 5 categories | - Very strong odor |
|  |  | - Noticeable odor |
|  |  | - Moderate |
|  |  | - Little or no odor |
|  |  | - No odor |
| Scalp odor after shampooing | 2 categories | - Yes |
|  |  | - No |
| Time to scalp oiliness after shampooing | 6 categories | - ≤4 hours |
|  |  | - 4–<8 hours |
|  |  | - 8–<12 hours |
|  |  | - 12–24 hours |
|  |  | - 1–2 days |
|  |  | - ≥2 days |
| Shampoo frequency | 4 categories | - ≥2 times per day |
|  |  | - Once per day |
|  |  | - Once every 2–3 days |
|  |  | - Once every ≥4 days |
| Hair drying frequency | 3 categories | - ≥5 times per week |
|  |  | - 1–4 times per week |
|  |  | - Less than once per week |
| Degree of hair drying | 5 categories | - 100% dried |
|  |  | - 80–90% dried |
|  |  | - 50–70% dried |
|  |  | - 10–40% dried |
|  |  | - Air-dried |
| Hair drying method | 3 categories | - - Air-dried |
|  |  | - - Hair dryer |
|  |  | - - Dyson Airwrap |
| Hair combing frequency | 3 categories | - ≥5 times per week |
|  |  | - 1–4 times per week |
|  |  | - Less than once per week |
| Hair tying frequency | 4 categories | - ≥5 times per week |
|  |  | - 1–4 times per week |
|  |  | - Less than once per week |
|  |  | - Male |
| Hair bleaching frequency | 3 categories | - ≥1 time every 6 weeks |
|  |  | - Once every 6 weeks to 3 months |
|  |  | - Less than once every 3 months |
| Hair dyeing frequency | 3 categories | - ≥1 time every 6 weeks |
|  |  | - Once every 6 weeks to 3 months |
|  |  | - Less than once every 3 months |
| Hair perming frequency | 3 categories | - ≥1 time every 6 weeks |
|  |  | - Once every 6 weeks to 3 months |
|  |  | - Less than once every 3 months |
| Haircut frequency | 3 categories | - ≥1 time every 6 weeks |
|  |  | - Once every 6 weeks to 3 months |
|  |  | - Less than once every 3 months |
| Use of functional hair/scalp products | 2 categories | - Yes |
|  |  | - No |
| Intention to use leave-on products | 2 categories | - Yes |
|  |  | - No |
| Importance of scalp and hair care | 5 categories | - Strongly agree |
|  |  | - Agree |
|  |  | - Neutral |
|  |  | - Disagree |
|  |  | - Strongly disagree |
| Proportion of cosmetic expenditure | 5 categories | - 0–10% |
|  |  | - 10–30% |
|  |  | - 30–50% |
|  |  | - 50–70% |
|  |  | - 80–100% |
| Alcohol consumption amount | 4 categories | - Once per week |
|  |  | - 2–3 times per week |
|  |  | - ≥4 times per week |
|  |  | - No alcohol consumption |
| Perceived stress level | 4 categories | - None |
|  |  | - Mild |
|  |  | - Moderate |
|  |  | - Severe |
| Timing of childbirth | 4 categories | - Within 1 year |
|  |  | - More than 1 year ago |
|  |  | - No experience |
|  |  | - Male |
| Menopausal status/timing | 6 categories | - Within 1 month |
|  |  | - Within 6 months |
|  |  | - Within 1 year |
|  |  | - More than 1 year |
|  |  | - No experience |
|  |  | - Male |

*Abbreviation*: BMI, body mass index; DBP, diastolic blood pressure; SBP, Systolic blood pressure.

## **Table S2.** Baseline characteristics of the study population in the discovery cohort from Korea and external validation cohorts of white and black participants.

|  | **Discovery cohort (Korean; n=305)** | **External validation cohort**  **(White; n=29)** | **External validation cohort (Black; n=29)** |
| --- | --- | --- | --- |
| **Sex, n (%)** |  |  |  |
| Male | 259 (84.92) | 21 (72.41) | 21 (72.41) |
| Female | 46 (15.08) | 8 (27.59) | 8 (27.59) |
| **Demographic and physiological characteristics, mean (SD)** |  |  |  |
| Age, years | 49.92 (11.94) | 35.86 (9.68) | 35.00 (8.01) |
| Height | 161.26 (7.16) | 168.03 (8.81) | 169.91 (8.08) |
| Weight | 61.25 (11.36) | 59.72 (10.91) | 70.64 (14.51) |
| BMI | 23.60 (4.49) | 21.07 (2.92) | 24.51 (4.93) |
| SBP | 118.53 (15.87) | 108.86 (15.57) | 118.38 (16.11) |
| DBP | 79.38 (10.55) | 73.72 (9.27) | 78.17 (10.42) |
| Pulse | 73.22 (10.83) | 76.55 (10.75) | 72.52 (10.12) |
| **Alopecia status, n (%)** |  |  |  |
| No suspected alopecia | 41 (13.44) | 3 (10.34) | 8 (27.59) |
| Suspected alopecia | 129 (42.30) | 9 (31.03) | 8 (27.59) |
| Currently experiencing alopecia | 76 (24.92) | 13 (44.83) | 7 (24.14) |
| Uncertain | 59 (19.34) | 4 (13.79) | 6 (20.69) |
| **Scalp type, n (%)** |  |  |  |
| Oily | 72 (23.61) | 5 (17.24) | 0 (0.00) |
| Normal | 94 (30.82) | 4 (13.79) | 7 (24.14) |
| Dry | 93 (30.49) | 9 (31.03) | 14 (48.28) |
| Not sure | 46 (15.08) | 11 (37.93) | 8 (27.59) |
| **Severity of scalp odor, n (%)** |  |  |  |
| Very strong | 5 (1.64) | 2 (6.90) | 0 (0.00) |
| Noticeable | 80 (26.23) | 1 (3.45) | 4 (13.79) |
| Moderate | 152 (49.84) | 6 (20.69) | 6 (20.69) |
| Little or no | 51 (16.72) | 6 (20.69) | 6 (20.69) |
| No | 17 (5.57) | 14 (48.28) | 13 (44.83) |
| **Scalp odor after shampooing, n (%)** |  |  |  |
| Yes | 62 (20.33) | 0 (0.00) | 5 (17.24) |
| No | 243 (79.67) | 29 (100.00) | 24 (82.76) |
| **Time to scalp oiliness after shampooing, n (%)** |  |  |  |
| ≤4 hours | 15 (4.92) | 4 (13.79) | 5 (17.24) |
| 4–<8 hours | 23 (7.54) | 5 (17.24) | 2 (6.90) |
| 8–<12 hours | 45 (14.75) | 2 (6.90) | 1 (3.45) |
| 12–24 hours | 58 (19.02) | 2 (6.90) | 2 (6.90) |
| 1–2 days | 132 (43.28) | 16 (55.17) | 19 (65.52) |
| ≥2 days | 32 (10.16) | 0 (0.00) | 0 (0.00) |
| **Shampoo frequency, n (%)** |  |  |  |
| ≥2 times per day | 15 (4.92) | 1 (3.45) | 5 (17.24) |
| Once per day | 225 (73.77) | 7 (24.14) | 7 (24.14) |
| Once every 2–3 days | 61 (20.00) | 16 (55.17) | 16 (55.17) |
| Once every ≥4 days | 4 (1.31) | 5 (17.24) | 1 (3.45) |
| **Hair drying frequency, n (%)** |  |  |  |
| ≥5 times per week | 155 (50.82) | 6 (20.69) | 0 (0.00) |
| 1–4 times per week | 78 (25.57) | 12 (41.38) | 6 (20.69) |
| Less than once per week | 72 (23.61) | 11 (37.93) | 23 (79.31) |
| **Degree of hair drying, n (%)** |  |  |  |
| 100% dried | 46 (15.08) | 5 (17.24) | 4 (13.79) |
| 80–90% dried | 152 (49.84) | 9 (31.03) | 7 (24.14) |
| 50–70% dried | 56 (18.36) | 4 (13.79) | 3 (10.34) |
| 10–40% dried | 13 (4.26) | 2 (6.90) | 2 (6.90) |
| Air-dried | 38 (12.46) | 9 (31.03) | 13 (44.83) |
| **Hair drying method, n (%)** |  |  |  |
| Air-dried | 60 (19.67) | 12 (41.38) | 16 (55.17) |
| Hair dryer | 232 (76.07) | 15 (51.72) | 10 (34.48) |
| Dyson Airwrap | 13 (4.26) | 2 (6.90) | 3 (10.34) |
| **Hair combing frequency, n (%)** |  |  |  |
| ≥5 times per week | 181 (59.34) | 25 (86.21) | 9 (31.03) |
| 1–4 times per week | 72 (23.61) | 1 (3.45) | 10 (34.48) |
| Less than once per week | 52 (17.05) | 3 (10.34) | 10 (34.48) |
| **Hair tying frequency, n (%)** |  |  |  |
| ≥5 times per week | 94 (30.82) | 8 (27.59) | 4 (13.79) |
| 1–4 times per week | 61 (20.00) | 6 (20.69) | 7 (24.14) |
| Less than once per week | 103 (33.77) | 7 (24.14) | 10 (34.48) |
| Male | 47 (15.41) | 8 (27.59) | 8 (27.59) |
| **Hair bleaching frequency, n (%)** |  |  |  |
| ≥1 time every 6 weeks | 14 (4.59) | 3 (10.34) | 0 (0.00) |
| Once every 6 weeks to 3 months | 18 (5.90) | 26 (89.66) | 3 (10.34) |
| Less than once every 3 months | 273 (89.51) | 0 (0.00) | 26 (89.66) |
| **Hair dyeing frequency, n (%)** |  |  |  |
| ≥1 time every 6 weeks | 53 (17.38) | 1 (3.45) | 1 (3.45) |
| Once every 6 weeks to 3 months | 72 (23.61) | 5 (17.24) | 3 (10.34) |
| Less than once every 3 months | 180 (59.02) | 23 (79.31) | 25 (86.21) |
| **Hair perming frequency, n (%)** |  |  |  |
| ≥1 time every 6 weeks | 10 (3.28) | 0 (0.00) | 1 (3.45) |
| Once every 6 weeks to 3 months | 32 (10.49) | 2 (6.90) | 1 (3.45) |
| Less than once every 3 months | 263 (86.23) | 27 (93.10) | 27 (93.10) |
| **Haircut frequency, n (%)** |  |  |  |
| ≥1 time every 6 weeks | 52 (17.05) | 2 (6.90) | 3 (10.34) |
| Once every 6 weeks to 3 months | 100 (32.79) | 12 (41.38) | 4 (13.79) |
| Less than once every 3 months | 153 (50.16) | 15 (51.72) | 22 (75.86) |
| **Use of functional hair/scalp products, n (%)** |  |  |  |
| Yes | 163 (53.44) | 18 (62.07) | 18 (62.07) |
| No | 142 (46.56) | 11 (37.93) | 11 (37.93) |
| **Intention to use leave-on products, n (%)** |  |  |  |
| Yes | 265 (86.89) | 26 (89.66) | 26 (89.66) |
| No | 40 (13.12) | 3 (10.34) | 3 (10.34) |
| **Importance of scalp and hair care, n (%)** |  |  |  |
| Strongly agree | 113 (37.05) | 14 (48.28) | 14 (48.28) |
| Agree | 148 (48.52) | 12 (41.38) | 11 (37.93) |
| Neutral | 42 (13.77) | 3 (10.34) | 3 (10.34) |
| Disagree | 0 (0.00) | 0 (0.00) | 0 (0.00) |
| Strongly disagree | 2 (0.66) | 0 (0.00) | 1 (3.45) |
| **Proportion of cosmetic expenditure, n (%)** |  |  |  |
| 0–10% | 93 (30.49) | 10 (34.48) | 10 (34.48) |
| 10–30% | 145 (47.54) | 12 (41.38) | 12 (41.38) |
| 30–50% | 53 (17.38) | 5 (17.24) | 5 (17.24) |
| 50–70% | 13 (4.26) | 2 (6.90) | 1 (3.45) |
| 80–100% | 1 (0.33) | 0 (0.00) | 1 (3.45) |
| **Alcohol consumption amount, n (%)** |  |  |  |
| Once per week | 71 (23.28) | 13 (44.83) | 8 (27.59) |
| 2–3 times per week | 23 (7.54) | 5 (17.24) | 1 (3.45) |
| ≥4 times per week | 12 (3.93) | 11 (37.93) | 2 (6.90) |
| No alcohol consumption | 199 (64.59) | 0 (0.00) | 18 (62.07) |
| **Perceived stress level, n (%)** |  |  |  |
| None | 10 (3.28) | 0 (0.00) | 2 (6.90) |
| Mild | 223 (73.11) | 8 (27.59) | 8 (27.59) |
| Moderate | 59 (19.34) | 14 (48.28) | 16 (55.17) |
| Severe | 13 (4.26) | 7 (24.14) | 3 (10.34) |
| **Timing of childbirth, n (%)** |  |  |  |
| Within 1 year | 5 (1.64) | 4 (13.79) | 1 (3.45) |
| More than 1 year ago | 201 (65.90) | 17 (58.62) | 9 (31.03) |
| Never | 52 (17.05) | 8 (27.59) | 12 (41.38) |
| Male | 47 (15.41) | 0 (0.00) | 7 (24.14) |
| **Menopausal status/timing, n (%)** |  |  |  |
| Within 1 month | 4 (1.31) | 2 (6.90) | 0 (0.00) |
| Within 6 months | 5 (1.64) | 0 (0.00) | 0 (0.00) |
| Within 1 year | 14 (4.59) | 0 (0.00) | 1 (3.45) |
| More than 1 year | 117 (38.36) | 19 (65.52) | 21 (72.41) |
| Never | 118 (38.69) | 8 (27.59) | 0 (0.00) |
| Male | 47 (15.41) | 0 (0.00) | 7 (24.14) |

*Abbreviations*: BMI, body mass index; SBP, systolic blood pressure; DBP, diastolic blood pressure; SD, Standard deviation.

## **Table S3.** A comparative analysis of the predictive performance of models using training and test datasets from Korean and external validation datasets from White and Black participants.

|  |  | **Any hair condition ^a^** | | **Mean ^b^** | | **SSCI** | | | | **Dryness** | | **Oiliness** | | **Erythema** | | **Dandruff** | |
| --- | --- | --- | --- | --- | --- | --- | --- | --- | --- | --- | --- | --- | --- | --- | --- | --- | --- |
|  | **Model** | AUROC | AUPRC | AUROC | AUPRC | MAE | RMSE | QWK | Spearman | AUROC | AUPRC | AUROC | AUPRC | AUROC | AUPRC | AUROC | AUPRC |
| **Korean**  **(Train), mean (95% CI)** | **Meta dataset** | 0.533 (0.473-0.593) | 0.450 (0.390-0.532) | 0.509 (0.434-0.588) | 0.169 (0.130-0.221) | 0.79 (0.73-0.85) | 0.99 (0.94-1.05) | 0.08 (-0.01-0.16) | 0.09 (-0.02-0.14) | 0.538 (0.364-0.700) | 0.152 (0.031-0.284) | 0.508 (0.447-0.571) | 0.325 (0.263-0.398) | 0.472 (0.246-0.697) | 0.053 (0.017-0.143) | 0.519 (0.442-0.590) | 0.144 (0.109-0.192) |
|  | **Image dataset** | 0.742 (0.691-0.790) | 0.694 (0.616-0.770) | 0.789 (0.749-0.830) | 0.460 (0.357-0.552) | 0.87 (0.80-0.95) | 1.14 (1.10-1.20) | 0.09 (-0.01-0.19) | 0.10 (-0.01-0.20) | 0.721 (0.600-0.835) | 0.196 (0.054-0.370) | 0.714 (0.657-0.771) | 0.526 (0.432-0.613) | 0.977 (0.952-0.996) | 0.631 (0.317-0.917) | 0.746 (0.661-0.827) | 0.488 (0.340-0.613) |
|  | **Microbiome dataset** | 0.558 (0.498-0.619) | 0.482 (0.407-0.560) | 0.513 (0.444-0.579) | 0.167 (0.136-0.208) | 0.87 (0.82-0.93) | 1.04 (0.99-1.09) | -0.10 (-0.18--0.02) | -0.13 (-0.24--0.02) | 0.500 (0.355-0.645) | 0.098 (0.030-0.218) | 0.546 (0.480-0.613) | 0.351 (0.282-0.430) | 0.455 (0.248-0.670) | 0.039 (0.018-0.057) | 0.551 (0.473-0.626) | 0.178 (0.118-0.244) |
|  | **Lipidomic dataset** | 0.827 (0.782-0.872) | 0.842 (0.793-0.885) | 0.713 (0.657-0.771) | 0.323 (0.274-0.371) | 0.81 (0.75-0.87) | 1.00 (0.95-1.05) | 0.05 (-0.04-0.13) | 0.06 (-0.5-0.16) | 0.790 (0.719-0.857) | 0.176 (0.064-0.301) | 0.725 (0.669-0.782) | 0.566 (0.471-0.650) | 0.492 (0.304-0.688) | 0.043 (0.020-0.062) | 0.839 (0.784-0.897) | 0.505 (0.358-0.635) |
|  | **Fusion** | 0.867 (0.827-0.904) | 0.867 (0.822-0.906) | 0.742 (0.689-0.803) | 0.312 (0.277-0.350) | 0.81 (0.74-0.88) | 1.06 (0.10-1.12) | 0.02 (-0.07-0.11) | 0.03 (-0.07-0.14) | 0.852 (0.798-0.901) | 0.169 (0.091-0.249) | 0.740 (0.687-0.792) | 0.551 (0.450-0.642) | 0.507 (0.324-0.704) | 0.043 (0.019-0.063) | 0.867 (0.823-0.912) | 0.486 (0.347-0.612) |
|  | **Meta + Image** | 0.807 (0.761-0.851) | 0.800 (0.744-0.850) | 0.898 (0.872-0.923) | 0.696 (0.596-0.781) | 0.46 (0.39-0.52) | 0.81 (0.73-0.88) | 0.53 (0.44-0.61) | 0.56 (0.47-0.64) | 0.943 (0.887-0.991) | 0.605 (0.339-0.844) | 0.753 (0.699-0.808) | 0.586 (0.490-0.677) | 0.964 (0.911-0.999) | 0.823 (0.532-1.000) | 0.931 (0.895-0.974) | 0.771 (0.652-0.871) |
|  | **Meta + Fusion** | 0.828 (0.784-0.871) | 0.802 (0.739-0.857) | 0.741 (0.691-0.798) | 0.315 (0.267-0.366) | 0.47 (0.41-0.54) | 0.81 (0.74-0.88) | 0.51 (0.43-0.60) | 0.54 (0.45-0.62) | 0.689 (0.540-0.845) | 0.129 (0.047-0.212) | 0.745 (0.692-0.796) | 0.570 (0.471-0.666) | 0.718 (0.568-0.869) | 0.115 (0.033-0.228) | 0.809 (0.748-0.874) | 0.440 (0.308-0.570) |
|  | **Image + Fusion** | 0.798 (0.752-0.844) | 0.775 (0.710-0.830) | 0.871 (0.831-0.910) | 0.636 (0.513-0.750) | 0.83 (0.76-0.89) | 1.05 (0.99-1.11) | 0.06 (-0.02-0.15) | 0.08 (-0.02-0.18) | 0.901 (0.814-0.980) | 0.524 (0.231-0.754) | 0.743 (0.690-0.798) | 0.573 (0.472-0.662) | 0.917 (0.821-0.999) | 0.666 (0.296-0.999) | 0.926 (0.885-0.974) | 0.790 (0.685-0.888) |
|  | **Meta + Image + Fusion** | 0.788 (0.742-0.836) | 0.766 (0.703-0.819) | 0.893 (0.861-0.922) | 0.676 (0.568-0.764) | 0.47 (0.40-0.53) | 0.81 (0.73-0.88) | 0.53 (0.45-0.62) | 0.55 (0.47-0.63) | 0.918 (0.843-0.983) | 0.537 (0.249-0.753) | 0.771 (0.719-0.823) | 0.612 (0.514-0.703) | 0.958 (0.898-0.999) | 0.778 (0.495-0.999) | 0.925 (0.884-0.971) | 0.779 (0.662-0.884) |
| **Korean**  **(Test)** | **Meta only** | 0.626 | 0.600 | 0.617 | 0.254 | 0.80 | 1.03 | 0.04 | 0.05 | 0.777 | 0.349 | 0.582 | 0.339 | 0.505 | 0.113 | 0.603 | 0.214 |
|  | **Image only** | 0.861 | 0.879 | 0.79 | 0.463 | 0.91 | 1.20 | 0.00 | 0.00 | 0.832 | 0.299 | 0.688 | 0.370 | 0.866 | 0.689 | 0.774 | 0.494 |
|  | **Microbiome** | 0.586 | 0.623 | 0.563 | 0.256 | 0.89 | 1.05 | -0.1 | -0.14 | 0.570 | 0.254 | 0.621 | 0.390 | 0.442 | 0.096 | 0.618 | 0.285 |
|  | **Lipidomic** | 0.889 | 0.916 | 0.719 | 0.314 | 0.82 | 1.01 | 0.04 | 0.02 | 0.805 | 0.322 | 0.715 | 0.446 | 0.570 | 0.150 | 0.787 | 0.338 |
|  | **Fusion** | 0.926 | 0.940 | 0.727 | 0.347 | 0.88 | 1.06 | -0.06 | -0.08 | 0.817 | 0.34 | 0.746 | 0.549 | 0.526 | 0.145 | 0.82 | 0.353 |
|  | **Meta + Image** | 0.903 | 0.904 | 0.907 | 0.765 | 0.37 | 0.75 | 0.64 | 0.65 | 0.98 | 0.892 | 0.752 | 0.576 | 0.956 | 0.913 | 0.94 | 0.68 |
|  | **Meta + Fusion** | 0.906 | 0.903 | 0.749 | 0.379 | 0.36 | 0.73 | 0.66 | 0.66 | 0.754 | 0.294 | 0.757 | 0.523 | 0.631 | 0.235 | 0.854 | 0.463 |
|  | **Image + Fusion** | 0.91 | 0.908 | 0.908 | 0.778 | 0.80 | 0.98 | 0.11 | 0.13 | 0.966 | 0.844 | 0.761 | 0.603 | 0.967 | 0.918 | 0.94 | 0.745 |
|  | **Meta + Image + Fusion** | 0.928 | 0.927 | 0.919 | 0.802 | 0.36 | 0.75 | 0.65 | 0.66 | 0.973 | 0.888 | 0.791 | 0.658 | 0.971 | 0.921 | 0.943 | 0.74 |
| **White** | **Meta + Image + Fusion** | 0.708 | 0.696 | 0.741 | 0.587 | 0.82 | 1.15 | 0.00 | 0.00 | - | - | 0.678 | 0.507 | 0.598 | 0.442 | 0.947 | 0.813 |
| **Black** | **Meta + Image + Fusion** | 0.581 | 0.688 | 0.648 | 0.674 | 0.90 | 1.20 | 0.10 | 0.09 | - | - | 0.505 | 0.626 | - | - | 0.792 | 0.722 |

Fusion indicates the integration of microbiome and lipidomic data. Blue highlight denotes the final selected model. CIs were estimated using bootstrap resampling (2,000 iterations).

a. **Any hair condition** indicates the presence of ≥1 hair-related condition.

b. **Mean** represents the average performance across all four hair-related outcomes.

*Abbreviations*: AUPRC, area under the precision-recall curve; AUROC, area under the receiver operating characteristic curve; CIs, confidence intervals; MAE, mean absolute error; QWK, quadratic weighted kappa; RMSE, root mean squared error; SSCI, Social Stigma Clinical Index.

## **Table S4.** Mapping of fusion latent variables to original microbiome and lipid features for hair-related outcome.

| **Latent** | **Feature** | **Modality** | **Correlation** |
| --- | --- | --- | --- |
| FUSPAP_001 | d__Bacteria;p__Bacillota_A;c__Clostridia;o__Tissierellales;f__Peptoniphilaceae;g__Anaerococcus;s__Anaerococcus octavius | Microbiome | 0.51 |
|  | d__Bacteria;p__Deinococcota;c__Deinococci;o__Deinococcales;f__Deinococcaceae;g__Deinococcus;__ | Microbiome | 0.48 |
|  | d__Bacteria;p__Pseudomonadota;c__Alphaproteobacteria;o__Rhizobiales;f__Rhizobiaceae;g__Agrobacterium;__ | Microbiome | -0.47 |
|  | d__Bacteria;p__Bacillota_A;c__Clostridia;o__Tissierellales;f__Peptoniphilaceae;g__Anaerococcus;__ | Microbiome | 0.43 |
|  | Phosphatidyl serine_16:0-22:6 | Lipidomic | 0.41 |
|  | d__Bacteria;p__Bacillota;c__Bacilli;o__Lactobacillales;f__Lactobacillaceae;g__Lactobacillus;s__Lactobacillus iners | Microbiome | -0.41 |
|  | Diacylglycerol_16:0-20:4 | Lipidomic | -0.4 |
|  | Phosphatidic acid_14:0-18:0 PA | Lipidomic | -0.39 |
|  | Diacylglycerol_16:0-18:1 | Lipidomic | -0.38 |
|  | Diacylglycerol_18:1-18:1 | Lipidomic | 0.38 |
|  | d__Bacteria;p__Actinomycetota;c__Actinomycetes;o__Actinomycetales;f__Micrococcaceae;g__Rothia;s__Rothia amarae | Microbiome | 0.38 |
|  | Phosphatidyl inositol_16:0-22:6 | Lipidomic | 0.38 |
|  | Phosphatidyl serine_18:1-18:1 | Lipidomic | 0.38 |
|  | Phosphatidic acid_18:0-18:2 PA | Lipidomic | 0.37 |
|  | d__Bacteria;p__Bacillota;c__Bacilli;o__Lactobacillales;f__Lactobacillaceae;g__Lactobacillus;__ | Microbiome | -0.37 |
| FUSPAP_008 | Fatty aldehye_2-nonenal | Lipidomic | -0.42 |
|  | Phosphatidyl inositol_16:0-18:2 | Lipidomic | -0.37 |
|  | d__Bacteria;p__Bacillota;c__Bacilli;o__Lactobacillales;f__Streptococcaceae;g__Streptococcus;__ | Microbiome | 0.36 |
|  | d__Bacteria;p__Pseudomonadota;c__Gammaproteobacteria;o__Pseudomonadales;f__Pseudomonadaceae;g__Pseudomonas_E;__ | Microbiome | 0.35 |
|  | Triglyceride_TG(44:1) | Lipidomic | 0.33 |
|  | Phosphatidyl choline_18:0-18:2 | Lipidomic | 0.32 |
|  | Phosphatidyl inositol_18:0-18:1 | Lipidomic | -0.32 |
|  | Wax ester_C30:0 | Lipidomic | -0.32 |
|  | Triglyceride_TG(48:4) | Lipidomic | 0.32 |
|  | Wax ester_C34:0 | Lipidomic | -0.31 |
|  | Triglyceride_TG(44:2) | Lipidomic | 0.31 |
|  | d__Bacteria;p__Bacillota_A;c__Clostridia;o__Tissierellales;f__Peptoniphilaceae;g__Anaerococcus;__ | Microbiome | -0.31 |
|  | Wax ester_C28:1 | Lipidomic | -0.31 |
|  | Phosphatidic acid_14:0-18:1 PA | Lipidomic | -0.31 |
|  | Wax ester_C38:0 | Lipidomic | -0.31 |
| FUSPAP_009 | Triglyceride_TG(52:2) | Lipidomic | -0.48 |
|  | Triglyceride_TG(54:0) | Lipidomic | -0.46 |
|  | Triglyceride_TG(52:5) | Lipidomic | -0.44 |
|  | Triglyceride_TG(54:3) | Lipidomic | -0.43 |
|  | d__Bacteria;p__Pseudomonadota;c__Alphaproteobacteria;o__Rhizobiales;f__Xanthobacteraceae;g__Bradyrhizobium;__ | Microbiome | -0.43 |
|  | Triglyceride_TG(52:4) | Lipidomic | -0.4 |
|  | Triglyceride_TG(53:3) | Lipidomic | -0.39 |
|  | Triglyceride_TG(54:1) | Lipidomic | -0.36 |
|  | Triglyceride_TG(53:4) | Lipidomic | -0.35 |
|  | Triglyceride_TG(54:2) | Lipidomic | -0.35 |
|  | Triglyceride_TG(51:5) | Lipidomic | -0.35 |
|  | d__Bacteria;p__Pseudomonadota;c__Gammaproteobacteria;o__Pseudomonadales;f__Pseudomonadaceae;g__Pseudomonas_E;__ | Microbiome | -0.34 |
|  | Triglyceride_TG(52:3) | Lipidomic | -0.32 |
|  | Triglyceride_TG (53:5) | Lipidomic | -0.32 |
|  | Triglyceride_TG(52:0) | Lipidomic | -0.32 |
| FUSPAP_010 | d__Bacteria;p__Actinomycetota;c__Coriobacteriia;o__Coriobacteriales;f__Coriobacteriaceae;g__Collinsella;__ | Microbiome | -0.53 |
|  | Triglyceride_TG(57:2) | Lipidomic | 0.53 |
|  | Triglyceride_TG(58:2) | Lipidomic | 0.5 |
|  | Phosphatidyl choline_16:0-22:6 | Lipidomic | -0.49 |
|  | Triglyceride_TG(57:1) | Lipidomic | 0.49 |
|  | Triglyceride_TG(58:3) | Lipidomic | 0.49 |
|  | Phosphatidic acid_14:0-18:1 PA | Lipidomic | 0.48 |
|  | Triglyceride_TG(58:1) | Lipidomic | 0.48 |
|  | Triglyceride_TG(58:0) | Lipidomic | 0.47 |
|  | Phosphatidyl choline_18:0-18:2 | Lipidomic | -0.47 |
|  | Fatty acids_C18:2 | Lipidomic | -0.47 |
|  | Fatty acids_Poly saturated FFA | Lipidomic | -0.47 |
|  | Triglyceride_TG(56:4) | Lipidomic | 0.46 |
|  | Phosphatidyl choline_18:0-22:6 | Lipidomic | -0.46 |
|  | Triglyceride_TG(56:5) | Lipidomic | 0.46 |
| FUSPAP_016 | Diacylglycerol_C16 | Lipidomic | -0.5 |
|  | Phosphatidyl inositol_16:0-18:0 | Lipidomic | -0.45 |
|  | Sphingomyelin_C22 | Lipidomic | 0.45 |
|  | Phosphatidyl inositol_14:0-16:0 | Lipidomic | -0.44 |
|  | d__Bacteria;p__Bacillota_A;c__Clostridia;o__Lachnospirales;f__Lachnospiraceae;g__Blautia;s__Blautia hansenii | Microbiome | 0.4 |
|  | Diacylglycerol_14:0-18:0 | Lipidomic | -0.39 |
|  | Phosphatidic acid_18:0 PA | Lipidomic | -0.38 |
|  | Diacylglycerol_14:0-16:0 | Lipidomic | -0.38 |
|  | Phosphatidyl inositol_C14 | Lipidomic | -0.38 |
|  | d__Bacteria;p__Bacillota;c__Bacilli;o__Lactobacillales;f__Streptococcaceae;g__Streptococcus;__ | Microbiome | -0.38 |
|  | Phosphatidyl inositol_16:0-22:6 | Lipidomic | -0.38 |
|  | d__Bacteria;p__Bacillota_A;c__Clostridia;o__Lachnospirales;f__Lachnospiraceae;g__Faecalimonas;s__Faecalimonas umbilicata | Microbiome | 0.37 |
|  | d__Bacteria;p__Pseudomonadota;c__Gammaproteobacteria;o__Pseudomonadales;f__Moraxellaceae;g__Moraxella_A;__ | Microbiome | -0.37 |
|  | d__Bacteria;p__Pseudomonadota;c__Alphaproteobacteria;o__Caulobacterales;f__Caulobacteraceae;g__Brevundimonas;__ | Microbiome | -0.36 |
|  | d__Bacteria;p__Actinomycetota;c__Coriobacteriia;o__Coriobacteriales;f__Coriobacteriaceae;g__Collinsella;__ | Microbiome | 0.36 |
| FUSPAP_028 | Diacylglycerol_C16 | Lipidomic | 0.49 |
|  | Phosphatidyl inositol_14:0-16:0 | Lipidomic | 0.46 |
|  | Ceramide NP_C20 | Lipidomic | 0.43 |
|  | Phosphatidyl inositol_C14 | Lipidomic | 0.42 |
|  | Phosphatidyl inositol_16:0-22:6 | Lipidomic | 0.42 |
|  | Phosphatidyl inositol_18:0-18:2 | Lipidomic | 0.41 |
|  | Diacylglycerol_C18 | Lipidomic | 0.39 |
|  | Triglyceride_TG(40:1) | Lipidomic | -0.39 |
|  | Triglyceride_TG(44:2) | Lipidomic | -0.39 |
|  | Phosphatidic acid_22:6 PA | Lipidomic | 0.39 |
|  | Phosphatidyl choline_14:0-18:0 | Lipidomic | 0.38 |
|  | Triglyceride_TG(44:1) | Lipidomic | -0.38 |
|  | d__Bacteria;p__Pseudomonadota;c__Gammaproteobacteria;o__Burkholderiales;f__Burkholderiaceae;g__Ralstonia;__ | Microbiome | -0.37 |
|  | Phosphatidyl choline_18:0-16:0 | Lipidomic | -0.37 |
|  | Triglyceride_TG(48:2) | Lipidomic | -0.37 |
| FUSPAP_029 | Wax ester_C34:1 | Lipidomic | -0.43 |
|  | Wax ester_C36:0 | Lipidomic | -0.43 |
|  | Wax ester_C36:1 | Lipidomic | -0.43 |
|  | Wax ester_C34:0 | Lipidomic | -0.43 |
|  | Wax ester_C38:1 | Lipidomic | -0.42 |
|  | Wax ester_C32:1 | Lipidomic | -0.42 |
|  | Wax ester_C38:0 | Lipidomic | -0.42 |
|  | Wax ester_C40:0 | Lipidomic | -0.41 |
|  | d__Bacteria;p__Actinomycetota;c__Actinomycetes;o__Actinomycetales;f__Micrococcaceae;g__Rothia;s__Rothia amarae | Microbiome | 0.39 |
|  | Wax ester_C32:0 | Lipidomic | -0.38 |
|  | Wax ester_C40:1 | Lipidomic | -0.38 |
|  | Wax ester_C30:0 | Lipidomic | -0.37 |
|  | d__Bacteria;p__Pseudomonadota;c__Alphaproteobacteria;o__Acetobacterales;f__Acetobacteraceae;__;__ | Microbiome | 0.37 |
|  | Wax ester_C26:1 | Lipidomic | -0.36 |
|  | d__Bacteria;p__Bacillota;c__Bacilli;o__Staphylococcales;f__Staphylococcaceae;g__Staphylococcus;s__Staphylococcus caprae | Microbiome | 0.35 |
| FUSPAP_041 | Triglyceride_TG(54:5) | Lipidomic | -0.47 |
|  | Ceramide NDS_C16 | Lipidomic | 0.43 |
|  | Ceramide NDS_Short chain ceramide NDS | Lipidomic | 0.42 |
|  | Ceramide NDS_C18 | Lipidomic | 0.42 |
|  | Ceramide NDS_C14 | Lipidomic | 0.42 |
|  | Triglyceride_TG(52:4) | Lipidomic | -0.39 |
|  | Ceramide NDS_C22 | Lipidomic | 0.39 |
|  | Diacylglycerol_18:0-18:1 | Lipidomic | 0.38 |
|  | Ceramide NDS_C24:1 | Lipidomic | 0.38 |
|  | d__Bacteria;p__Actinomycetota;c__Actinomycetes;o__Actinomycetales;f__Micrococcaceae;g__Kocuria;__ | Microbiome | 0.37 |
|  | Ceramide NDS_Total ceramide NDS | Lipidomic | 0.37 |
|  | Phosphatidic acid_16:0 PA | Lipidomic | -0.37 |
|  | Ceramide NP_C18 | Lipidomic | 0.36 |
|  | Diacylglycerol_16:0-20:4 | Lipidomic | -0.36 |
|  | Ceramide NP_C24:1 | Lipidomic | 0.36 |
| FUSPAP_043 | Cholesterol_Cholesterol sulfate | Lipidomic | 0.49 |
|  | Sphingomyelin_C24 | Lipidomic | 0.48 |
|  | Sphingomyelin_C24:1 | Lipidomic | 0.47 |
|  | d__Bacteria;p__Pseudomonadota;c__Gammaproteobacteria;o__Pseudomonadales;f__Pseudomonadaceae;g__Pseudomonas_E;__ | Microbiome | -0.46 |
|  | Diacylglycerol_18:0-20:4 | Lipidomic | 0.45 |
|  | Sphingomyelin_C26 | Lipidomic | 0.45 |
|  | Phosphatidyl serine_C16 | Lipidomic | -0.45 |
|  | Phosphatidyl choline_18:0-16:0 | Lipidomic | -0.44 |
|  | Wax ester_C24:0 | Lipidomic | 0.44 |
|  | Ceramide NS_C14 | Lipidomic | 0.44 |
|  | Triglyceride_TG(49:2) | Lipidomic | -0.44 |
|  | Wax ester_C28:0 | Lipidomic | 0.43 |
|  | d__Bacteria;p__Bacillota;c__Bacilli;o__Lactobacillales;f__Lactobacillaceae;g__Lactobacillus;s__Lactobacillus iners | Microbiome | -0.42 |
|  | Triglyceride_TG(44:0) | Lipidomic | -0.42 |
|  | Triglyceride_TG(50:3) | Lipidomic | -0.42 |
| FUSPAP_046 | Phosphatidyl serine_18:1-18:1 | Lipidomic | 0.62 |
|  | Phosphatidic acid_14:0-18:0 PA | Lipidomic | -0.6 |
|  | Diacylglycerol_C18 | Lipidomic | 0.6 |
|  | Diacylglycerol_16:0-18:2 | Lipidomic | -0.59 |
|  | Ceramide NP_C24:1 | Lipidomic | 0.57 |
|  | Phosphatidic acid_18:0-18:2 PA | Lipidomic | 0.57 |
|  | Triglyceride_TG(49:1) | Lipidomic | -0.56 |
|  | Diacylglycerol_16:0-20:4 | Lipidomic | -0.56 |
|  | Diacylglycerol_18:0-14:0 | Lipidomic | -0.55 |
|  | Phosphatidyl choline_C14 | Lipidomic | 0.55 |
|  | Phosphatidyl serine_16:0-22:6 | Lipidomic | 0.55 |
|  | Diacylglycerol_18:0-22:6 | Lipidomic | 0.54 |
|  | Fatty acids_C20 | Lipidomic | -0.54 |
|  | Ceramide NP_Long chain ceramide NP | Lipidomic | 0.53 |
|  | Phosphatidic acid_14:0-16:0 PA | Lipidomic | -0.53 |

Variables prefixed with FUSPAP denote latent features derived through dimensionality reduction using the PAPRICA-based fusion autoencoder, capturing integrated patterns across microbiome and lipidomic data. To enhance interpretability, each latent variable was mapped to the original microbiome taxa and lipidomic components most strongly associated with it based on correlation analysis. The reported values represent the magnitude and direction of these associations.

## **Table S5.** Mapping of fusion latent variables to original microbiome and lipid features for SSCI outcome.

| Latent | Feature | Modality | Correlation |
| --- | --- | --- | --- |
| FUSPAP_005 | d__Bacteria;p__Actinomycetota;c__Actinomycetes;o__Actinomycetales;f__Dermatophilaceae;g__Dermacoccus;s__Dermacoccus nishinomiyaensis | Microbiome | 0.51 |
|  | Wax ester_C44:0 | Lipidomic | -0.45 |
|  | Wax ester_C42:1 | Lipidomic | -0.45 |
|  | Triglyceride_TG(40:0) | Lipidomic | 0.45 |
|  | Wax ester_C44:1 | Lipidomic | -0.45 |
|  | Wax ester_C42:0 | Lipidomic | -0.44 |
|  | Ceramide NP_C16 | Lipidomic | -0.43 |
|  | Triglyceride_TG(51:4) | Lipidomic | 0.42 |
|  | Phosphatidyl serine_18:0-18:2 | Lipidomic | 0.42 |
|  | Phosphatidyl inositol_16:0-18:1 | Lipidomic | -0.42 |
|  | Wax ester_C40:1 | Lipidomic | -0.41 |
|  | Triglyceride_TG(50:5) | Lipidomic | 0.4 |
|  | Fatty aldehye_C16 | Lipidomic | 0.39 |
|  | Ceramide EOS_d18:1/C30:1/C18:2 | Lipidomic | 0.39 |
|  | Fatty aldehye_C14 | Lipidomic | 0.39 |
| FUSPAP_021 | Triglyceride_TG(50:0) | Lipidomic | 0.54 |
|  | Phosphatidyl inositol_C18 | Lipidomic | -0.54 |
|  | Fatty aldehye_C20 | Lipidomic | 0.52 |
|  | Phosphatidyl choline_16:0-18:2 | Lipidomic | -0.52 |
|  | Phosphatidyl serine_16:0-14:0 | Lipidomic | 0.52 |
|  | Triglyceride_TG(50:1) | Lipidomic | 0.51 |
|  | Fatty acids_C18:1 | Lipidomic | 0.51 |
|  | Fatty acids_C22 | Lipidomic | 0.5 |
|  | Sphingomyelin_Total SM | Lipidomic | -0.5 |
|  | Phosphatidic acid_18:2 PA | Lipidomic | -0.5 |
|  | Ceramide EOS_d18:1/C32:1/C18:2 | Lipidomic | -0.5 |
|  | Sphingomyelin_C20 | Lipidomic | -0.49 |
|  | Phosphatidyl serine_14:0-16:0 | Lipidomic | 0.49 |
|  | Diacylglycerol_C18 | Lipidomic | 0.49 |
|  | Fatty acids_C18 | Lipidomic | 0.48 |
| FUSPAP_027 | Phosphatidyl serine_16:0-20:4 | Lipidomic | 0.48 |
|  | d__Bacteria;p__Pseudomonadota;c__Alphaproteobacteria;o__Sphingomonadales;f__Sphingomonadaceae;g__Sphingomonas;__ | Microbiome | -0.44 |
|  | Diacylglycerol_16:0-18:0 | Lipidomic | -0.44 |
|  | Diacylglycerol_16:0-18:1 | Lipidomic | -0.43 |
|  | d__Bacteria;p__Pseudomonadota;c__Alphaproteobacteria;o__Rhodobacterales;f__Rhodobacteraceae;g__Paracoccus;s__Paracoccus sphaerophysae | Microbiome | 0.41 |
|  | d__Bacteria;p__Bacillota_A;c__Clostridia;o__Lachnospirales;f__Lachnospiraceae;g__Blautia;s__Blautia hansenii | Microbiome | 0.4 |
|  | Phosphatidyl inositol_16:0-18:1 | Lipidomic | -0.39 |
|  | Ceramide NP_C14 | Lipidomic | 0.38 |
|  | d__Bacteria;p__Bacillota;c__Bacilli;o__Lactobacillales;f__Lactobacillaceae;g__Lactobacillus;s__Lactobacillus iners | Microbiome | -0.38 |
|  | Ceramide NS_C20 | Lipidomic | 0.38 |
|  | Diacylglycerol_16:0-20:4 | Lipidomic | -0.37 |
|  | Fatty acids_C16:1 | Lipidomic | -0.37 |
|  | Ceramide NS_C14 | Lipidomic | 0.36 |
|  | Cholesterol_Cholesterol | Lipidomic | 0.36 |
|  | Ceramide EOS_Total EOS | Lipidomic | 0.35 |
| FUSPAP_028 | Diacylglycerol_C16 | Lipidomic | 0.49 |
|  | Phosphatidyl inositol_14:0-16:0 | Lipidomic | 0.46 |
|  | Ceramide NP_C20 | Lipidomic | 0.43 |
|  | Phosphatidyl inositol_C14 | Lipidomic | 0.42 |
|  | Phosphatidyl inositol_16:0-22:6 | Lipidomic | 0.42 |
|  | Phosphatidyl inositol_18:0-18:2 | Lipidomic | 0.41 |
|  | Diacylglycerol_C18 | Lipidomic | 0.39 |
|  | Triglyceride_TG(40:1) | Lipidomic | -0.39 |
|  | Triglyceride_TG(44:2) | Lipidomic | -0.39 |
|  | Phosphatidic acid_22:6 PA | Lipidomic | 0.39 |
|  | Phosphatidyl choline_14:0-18:0 | Lipidomic | 0.38 |
|  | Triglyceride_TG(44:1) | Lipidomic | -0.38 |
|  | d__Bacteria;p__Pseudomonadota;c__Gammaproteobacteria;o__Burkholderiales;f__Burkholderiaceae;g__Ralstonia;__ | Microbiome | -0.37 |
|  | Phosphatidyl choline_18:0-16:0 | Lipidomic | -0.37 |
|  | Triglyceride_TG(48:2) | Lipidomic | -0.37 |
| FUSPAP_029 | Wax ester_C34:1 | Lipidomic | -0.43 |
|  | Wax ester_C36:0 | Lipidomic | -0.43 |
|  | Wax ester_C36:1 | Lipidomic | -0.43 |
|  | Wax ester_C34:0 | Lipidomic | -0.43 |
|  | Wax ester_C38:1 | Lipidomic | -0.42 |
|  | Wax ester_C32:1 | Lipidomic | -0.42 |
|  | Wax ester_C38:0 | Lipidomic | -0.42 |
|  | Wax ester_C40:0 | Lipidomic | -0.41 |
|  | d__Bacteria;p__Actinomycetota;c__Actinomycetes;o__Actinomycetales;f__Micrococcaceae;g__Rothia;s__Rothia amarae | Microbiome | 0.39 |
|  | Wax ester_C32:0 | Lipidomic | -0.38 |
|  | Wax ester_C40:1 | Lipidomic | -0.38 |
|  | Wax ester_C30:0 | Lipidomic | -0.37 |
|  | d__Bacteria;p__Pseudomonadota;c__Alphaproteobacteria;o__Acetobacterales;f__Acetobacteraceae;__;__ | Microbiome | 0.37 |
|  | Wax ester_C26:1 | Lipidomic | -0.36 |
|  | d__Bacteria;p__Bacillota;c__Bacilli;o__Staphylococcales;f__Staphylococcaceae;g__Staphylococcus;s__Staphylococcus caprae | Microbiome | 0.35 |
| FUSPAP_030 | Phosphatidyl choline_18:1-18:0 | Lipidomic | -0.55 |
|  | Phosphatidyl serine_16:0-14:0 | Lipidomic | -0.53 |
|  | Triglyceride_TG(50:0) | Lipidomic | -0.51 |
|  | Ceramide EOS_d18:1/C32:1/C18:2 | Lipidomic | 0.51 |
|  | Phosphatidyl serine_16:0-18:1 | Lipidomic | 0.49 |
|  | Phosphatidyl serine_18:0-14:0 | Lipidomic | -0.49 |
|  | Ceramide NS_Total ceramide NS | Lipidomic | -0.49 |
|  | Triglyceride_TG(50:1) | Lipidomic | -0.49 |
|  | Triglyceride_TG(51:3) | Lipidomic | 0.48 |
|  | Ceramide NS_C16 | Lipidomic | -0.48 |
|  | Ceramide NP_C22 | Lipidomic | -0.48 |
|  | Triglyceride_TG(48:3) | Lipidomic | 0.48 |
|  | Ceramide NS_Long chain ceramide NS | Lipidomic | -0.48 |
|  | Phosphatidyl choline_18:1-18:1 | Lipidomic | -0.48 |
|  | Diacylglycerol_16:0-22:6 | Lipidomic | 0.47 |
| FUSPAP_034 | Ceramide NP_C26 | Lipidomic | -0.55 |
|  | Triglyceride_TG(42:2) | Lipidomic | -0.55 |
|  | Fatty acids_Total FFA | Lipidomic | -0.48 |
|  | Fatty acids_Saturate FFA | Lipidomic | -0.47 |
|  | Triglyceride_TG(44:1) | Lipidomic | -0.47 |
|  | Sphingomyelin_C24 | Lipidomic | 0.47 |
|  | Fatty acids_C16 | Lipidomic | -0.46 |
|  | Fatty acids_C18:3 | Lipidomic | -0.46 |
|  | Phosphatidyl choline_18:0-18:1 | Lipidomic | -0.45 |
|  | Fatty aldehye_C18 | Lipidomic | 0.44 |
|  | Triglyceride_TG(46:5) | Lipidomic | -0.44 |
|  | Fatty acids_Mono saturated FFA | Lipidomic | -0.43 |
|  | Cholesterol_Cholesterol sulfate | Lipidomic | 0.43 |
|  | Cholesterol_7慣-hydroxy Cholesterol | Lipidomic | 0.43 |
|  | Triglyceride_TG(40:1) | Lipidomic | -0.42 |
| FUSPAP_036 | d__Bacteria;p__Pseudomonadota;c__Alphaproteobacteria;o__Rhizobiales;f__Xanthobacteraceae;g__Bradyrhizobium;__ | Microbiome | -0.45 |
|  | d__Bacteria;p__Bacillota_A;c__Clostridia;o__Tissierellales;f__Peptoniphilaceae;g__Anaerococcus;__ | Microbiome | 0.44 |
|  | d__Bacteria;p__Cyanobacteriota;c__Cyanobacteriia;o__Cyanobacteriales;f__Chroococcidiopsidaceae;__;__ | Microbiome | 0.39 |
|  | d__Bacteria;p__Bacillota;c__Bacilli;o__Lactobacillales;f__Enterococcaceae;g__Enterococcus_D;__ | Microbiome | 0.39 |
|  | d__Bacteria;p__Bacillota;c__Bacilli;__;__;__;__ | Microbiome | -0.37 |
|  | d__Bacteria;p__Bacillota;c__Bacilli;o__Lactobacillales;f__Lactobacillaceae;g__Lactiplantibacillus;__ | Microbiome | 0.35 |
|  | d__Bacteria;p__Bacillota;c__Bacilli;o__Lactobacillales;f__Streptococcaceae;g__Streptococcus;__ | Microbiome | -0.34 |
|  | d__Bacteria;p__Cyanobacteriota;c__Cyanobacteriia;o__Cyanobacteriales;f__Chroococcidiopsidaceae;g__Aliterella;s__Aliterella atlantica | Microbiome | 0.33 |
|  | d__Bacteria;p__Pseudomonadota;c__Alphaproteobacteria;o__Sphingomonadales;f__Sphingomonadaceae;g__Sphingomonas;__ | Microbiome | -0.33 |
|  | d__Bacteria;p__Bacillota;c__Bacilli;o__Lactobacillales;f__Lactobacillaceae;g__Lactobacillus;__ | Microbiome | -0.31 |
|  | d__Bacteria;p__Pseudomonadota;c__Gammaproteobacteria;o__Enterobacterales;f__Enterobacteriaceae;g__Pantoea;s__Pantoea sp020179835 | Microbiome | 0.31 |
|  | d__Bacteria;p__Pseudomonadota;c__Gammaproteobacteria;o__Pseudomonadales;f__Pseudomonadaceae;g__Pseudomonas_E;__ | Microbiome | -0.3 |
|  | Diacylglycerol_16:0-18:0 | Lipidomic | -0.3 |
|  | d__Bacteria;p__Bacillota;c__Bacilli;o__Lactobacillales;f__Lactobacillaceae;g__Lactobacillus;s__Lactobacillus iners | Microbiome | -0.29 |
|  | d__Bacteria;p__Pseudomonadota;c__Gammaproteobacteria;o__Burkholderiales;f__Burkholderiaceae;__;__ | Microbiome | 0.29 |
| FUSPAP_041 | Triglyceride_TG(54:5) | Lipidomic | -0.47 |
|  | Ceramide NDS_C16 | Lipidomic | 0.43 |
|  | Ceramide NDS_Short chain ceramide NDS | Lipidomic | 0.42 |
|  | Ceramide NDS_C18 | Lipidomic | 0.42 |
|  | Ceramide NDS_C14 | Lipidomic | 0.42 |
|  | Triglyceride_TG(52:4) | Lipidomic | -0.39 |
|  | Ceramide NDS_C22 | Lipidomic | 0.39 |
|  | Diacylglycerol_18:0-18:1 | Lipidomic | 0.38 |
|  | Ceramide NDS_C24:1 | Lipidomic | 0.38 |
|  | d__Bacteria;p__Actinomycetota;c__Actinomycetes;o__Actinomycetales;f__Micrococcaceae;g__Kocuria;__ | Microbiome | 0.37 |
|  | Ceramide NDS_Total ceramide NDS | Lipidomic | 0.37 |
|  | Phosphatidic acid_16:0 PA | Lipidomic | -0.37 |
|  | Ceramide NP_C18 | Lipidomic | 0.36 |
|  | Diacylglycerol_16:0-20:4 | Lipidomic | -0.36 |
|  | Ceramide NP_C24:1 | Lipidomic | 0.36 |
| FUSPAP_063 | Sphingoid bases_Sa | Lipidomic | -0.47 |
|  | Sphingomyelin_C26:1 | Lipidomic | -0.38 |
|  | Sphingoid bases_Sa1P | Lipidomic | -0.37 |
|  | Phosphatidyl choline_C16 | Lipidomic | 0.36 |
|  | Fatty acids_C24 | Lipidomic | 0.34 |
|  | Phosphatidyl choline_C14 | Lipidomic | 0.34 |
|  | Fatty acids_C24:1 | Lipidomic | 0.32 |
|  | d__Bacteria;p__Pseudomonadota;c__Gammaproteobacteria;o__Pseudomonadales;f__Pseudomonadaceae;g__Pseudomonas_E;__ | Microbiome | -0.32 |
|  | Ceramide NS_C26 | Lipidomic | 0.31 |
|  | d__Bacteria;p__Actinomycetota;c__Actinomycetes;o__Actinomycetales;f__Micrococcaceae;g__Kocuria;__ | Microbiome | 0.31 |
|  | Ceramide EOS_d18:1/C30:0/C18:2 | Lipidomic | 0.31 |
|  | Ceramide NS_C24:1 | Lipidomic | 0.31 |
|  | Phosphatidyl inositol_14:0-18:0 | Lipidomic | 0.31 |
|  | Diacylglycerol_18:1-18:1 | Lipidomic | 0.31 |
|  | Sphingoid bases_SPN | Lipidomic | -0.3 |

Variables prefixed with FUSPAP denote latent features derived through dimensionality reduction using the PAPRICA-based fusion autoencoder, capturing integrated patterns across microbiome and lipidomic data. To enhance interpretability, each latent variable was mapped to the original microbiome taxa and lipidomic components most strongly associated with it based on correlation analysis. The reported values represent the magnitude and direction of these associations.

# **4 Reference**

1.	Shin KO, Lee JH, Chae S, et al. Small EVs From Adipose-Derived MSCs Modulate Epidermal Barrier and Inflammation Via Sphingosine-1-Phosphate Signaling Pathway. *J Extracell Vesicles* 2025; **14**(7): e70121.

2.	J P, A S. Synergistic fusion: An integrated pipeline of CLAHE, YOLO models, and advanced super-resolution for enhanced thermal eye detection. *PLoS One* 2025; **20**(7): e0328227.

3.	Tran VT, Pham TH, Phan NKC. Cervical image visualization and improvement for clinical support in diagnosis and treatment. *Sci Rep* 2025; **15**(1): 41732.

4.	Nasr M, Piórkowski A, Brzostowski K, El-Samie FEA. A novel approach for CT image smoothing: Quaternion Bilateral Filtering for kernel conversion. *Comput Biol Med* 2025; **196**(Pt C): 110644.

5.	Sanabria-Mazo JP, Navarrete J, Serrat M, et al. Assessment of stigma in Spanish people with chronic pain using the 8-item Stigma Scale for Chronic Illnesses (SSCI-8). *J Pain* 2025; **37**: 105538.

6.	Si Y, Abdollahi A, Ashrafi N, et al. Optimized feature selection and advanced machine learning for stroke risk prediction in revascularized coronary artery disease patients. *BMC Med Inform Decis Mak* 2025; **25**(1): 276.

7.	Tavolara TE, Gurcan MN, Niazi MKK. Contrastive Multiple Instance Learning: An Unsupervised Framework for Learning Slide-Level Representations of Whole Slide Histopathology Images without Labels. *Cancers (Basel)* 2022; **14**(23).

8.	Sornapudi S, Singh R. Self-supervised backbone framework for diverse agricultural vision tasks. *arXiv preprint arXiv:240315248* 2024.

9.	Cho J, Woo S, Hwang SH, et al. A Multimodal Predictive Model for Chronic Kidney Disease and Its Association With Vascular Complications in Patients With Type 2 Diabetes: Model Development and Validation Study in South Korea and the U.K. *Diabetes Care* 2025; **48**(9): 1562–70.

10.	Novichkov PS, Chandonia JM, Arkin AP. CORAL: A framework for rigorous self-validated data modeling and integrative, reproducible data analysis. *Gigascience* 2022; **11**.

11.	Cao W, Mirjalili V, Raschka S. Rank consistent ordinal regression for neural networks with application to age estimation. *Pattern Recognition Letters* 2020; **140**: 325–31.

12.	Soenksen LR, Ma Y, Zeng C, et al. Integrated multimodal artificial intelligence framework for healthcare applications. *NPJ Digit Med* 2022; **5**(1): 149.

13.	Bamberger T, Consortium D, Borenstein E. Phenotype-driven parallel embedding for microbiome multi-omic data integration. *bioRxiv* 2025: 2025.12.05.692119.

14.	Kim S, Kim H, Kim S, et al. Machine Learning–Based Prediction of Substance Use in Adolescents in Three Independent Worldwide Cohorts: Algorithm Development and Validation Study. *J Med Internet Res* 2025; **27**: e62805.

15.	Bagheri S, Taridashti S, Farahani H, Watson P, Rezvani E. Multilayer perceptron modeling for social dysfunction prediction based on general health factors in an Iranian women sample. *Front Psychiatry* 2023; **14**: 1283095.

16.	Zhang Y, Ming W, Yu B, et al. Robust integration and annotation of single-cell and spatial omics data using interpretable gene programs. *Cell Genom* 2025: 101105.

17.	Armstrong ME, Cairns BJ, Green J, Reeves GK, Beral V. Reported frequency of physical activity in a large epidemiological study: relationship to specific activities and repeatability over time. *BMC Med Res Methodol* 2011; **11**: 97.

18.	Wang C, Zuo C, Su Z, et al. Deep Learning and Explainable AI: New Pathways to Genetic Insights. *arXiv preprint arXiv:250509873* 2025.
